# Efficient Ni/Au Mesh Transparent Electrodes for ITO-Free Planar Perovskite Solar Cells

**DOI:** 10.3390/nano9070932

**Published:** 2019-06-28

**Authors:** Dazheng Chen, Gang Fan, Hongxiao Zhang, Long Zhou, Weidong Zhu, He Xi, Hang Dong, Shangzheng Pang, Xiaoning He, Zhenhua Lin, Jincheng Zhang, Chunfu Zhang, Yue Hao

**Affiliations:** 1 Wide Bandgap Semiconductor Technology Disciplines State Key Laboratory, School of Microelectronics, Xidian University, Xi’an 710071, China; 2 Shaanxi Joint Key Laboratory of Graphene, Xidian University, Xi’an 710071, China; 3 School of Advanced Materials and Nanotechnology, Xidian University, Xi’an 710071, China

**Keywords:** metal mesh, transparent electrode, photolithography, perovskite solar cell, large-area solar cell

## Abstract

Indium thin oxide (ITO)-free planar perovskite solar cells (PSCs) were fabricated at a low temperature (150 °C) in this work based on the transparent electrode of photolithography processed nickel/gold (Ni/Au) mesh and the high conductivity polymer, PH1000. Ultrathin Au was introduced to increase the conductivity of metal mesh, and the optimal hexagonal Ni (30 nm)/Au (10 nm) mesh (line width of 5 μm) shows a transmittance close to 80% in the visible light region and a sheet resistance lower than 16.9 Ω/sq. The conductive polymer PH1000 not only smooths the raised surface of the metal mesh but also enhances the charge collection ability of metal mesh. The fabricated PSCs have the typical planar structure (glass/Ni-Au mesh/PH1000/PEDOT:PSS/MA_y_FA_1−y_PbI_x_Cl_3−x_/PCBM/BCP/Ag) and the champion PSC (0.09 cm^2^) obtains a power conversion efficiency (PCE) of 13.88%, negligible current hysteresis, steady current density and PCE outputs, and good process repeatability. Its photovoltaic performance and stability are comparable to the reference PSC based on the ITO electrodes (PCE = 15.70%), which demonstrates that the Ni/Au mesh transparent electrodes are a promising ITO alternative to fabricate efficient PSCs. The relatively lower performance of Ni/Au based PSC results from the relatively slower charge extraction and stronger charge recombination than the ITO based PSC. Further, we tried to fabricate the large area (1 cm^2^) device and achieve a PCE over 6% with negligible hysteresis and steady current density and PCE outputs. The improvements of perovskite film quality and interface modification should be an effective approach to further enhance the device performance of Ni/Au based PSCs, and the Ni/Au mesh electrode may find wider applications in PSCs and flexible devices.

## 1. Introduction

Organic-inorganic hybrid perovskites solar cells (PSCs) have attracted more and more attention due to their advantages of low fabrication cost, light weight, solution processability, tunable light absorption range, bipolar transport properties, large-area manufacturing, and compatibility to both rigid and flexible substrates. Their rapid progress of increased power conversion efficiency (PCEs) from 3.8% to 23.32% and improved stability indicate many potential applications, including in photovoltaic plants, photovoltaic curtains, building integrated photovoltaic materials, wearable electronics devices, and even space power systems [1,2,3,4,5,6,7,8,9,10]. And the efficient bottom transparent electrode is primary and crucial to meeting these versatile applications. Nowadays, transparent conductive oxides, such as the indium thin oxide (ITO) is the most commonly used transparent electrode in PSCs. However, ITO prices are becoming more and more expensive because of the rising cost of indium. On the other hand, the ITO shows a relatively large sheet resistance, brittleness, and poor mechanical robustness [11,12]. There will be cracks on ITO surface at a bending radius (<10 mm) against repeated bending, which will further decrease its conductivity and degrade the device performance, making it incompatible with low-cost technology, such as roll-to-roll printing [13].

To overcome the drawbacks of ITO, many alternatives have been developed with good electrical and optical properties, such as graphene [14,15], carbon nanotubes [16], metal nanowires [17,18], transparent conducting oxide nanocrystal [19], conducting polymer [20,21] ultrathin metal films [21,22,23,24], and metal-mesh transparent conductive electrodes (TCEs) [25,26]. Among the alternatives, metal meshes have excellent ductility and can be easily fabricated by mature evaporation process, which is more suitable for large-area device production. Metal-mesh TCEs are highly bendable as well, and silver metal-mesh TCEs have been successfully used to fabricate organic solar cells, PSCs, and flexible devices [5,27]. More importantly, the conductivity and transmittance of metal mesh can be adjusted by structure parameters such as the metal-grid pitch, line width, and film thickness. And there are many ways to achieve size-tunable metal-mesh TCEs, such as laser sintering of nanoparticle ink, lithographic patterning, grain boundary lithography, nanoimprint lithography, and photolithography [28,29,30,31]. Photolithography is a standard fabrication process in semiconductor devices and integrated circuits, which determines their feature size (≤7 nm) and operation performance. It is believed that the photolithography process provides more precise control of the shape and size of the objects it creates and can create patterns over an entire surface cost-effectively [32]. In addition, the photolithography related processes are low-temperature technology and compatible to large area substrate (18 inches) or flexible substrates. Therefore, photolithography is a promising process to prepare highly consistent metal mesh for transparent electrode and solar cell applications [33,34,35].

We have proved in our previous work [36] that the adhesion of Ag on a glass substrate is relatively poorer than that of Ni, and Ni mesh processed by photolithography has been successfully used to fabricate PSCs, but the device performance (PCE = 5.74%) should be further improved. In this work, we optimize the thickness and shape of Ni meshes, Au is used to increase the conductivity of metal mesh, the high conductivity polymer PH1000 is employed to smooth the raised surface of metal mesh and enhance the charge collection ability of metal mesh, the optimized two-step MA_y_FA_1-y_PbI_x_Cl_3-x_ perovskite is used as the light absorber, and the fabricated ITO free PSCs have a structure of Glass/hexagonal Ni (30 nm)-Au (10 nm) mesh/PH1000/PEDOT:PSS/MA_y_FA_1−y_PbI_x_Cl_3−x_/PCBM/BCP/Ag. The champion PSC (0.09 cm^2^) obtains a PCE of 13.88%, negligible current hysteresis, steady current density and PCE outputs, good repeatability and storing stability. Further, we tried to fabricate the large area (1 cm^2^) devices under the same process, and achieve a PCE over 6% with negligible hysteresis and stable steady-state outputs. The comparable performance to the ITO based reference PSC demonstrates that the Ni/Au mesh transparent electrodes are a promising ITO alternative to fabricate efficient PSCs. 

## 2. Materials and Methods

### 2.1. Materials

All materials and reagents, Methylammonium iodide (MAI, 99.8%, Dyesol, Queanbeyan, Australia), Formamidinium iodide (FAI, 99.8%, Dyesol, Queanbeyan, Australia), Lead iodide (PbI_2_, 99.999%, Sigma-Aldrich, Saint Louis, MI, USA), Lead chloride (PbCl_2_, 99.999%, Sigma-Aldrich, Saint Louis, MI, USA), Phenyl-C_61_-butyric acid methyl ester (PCBM, 98%, Nano-C, Westwood, MA, USA), Poly(3,4-ethy-lenedioxythiophene) poly(styrenesulfonate) (PEDOT:PSS, Clevios PVP Al 4083, Heraeus, Hanau, Germany), high conductivity polymer PH1000 (Clevios PH1000, Heraeus, Hanau, Germany), N,N’-Dimethylformamide (DMF, 99.8%, Aladdin, Beijing, China), Chlorobenzene (CB, 99.8%, Sigma-Aldrich, Saint Louis, MI, USA), Bathocuproine (BCP, 96%, Sigma-Aldrich, Saint Louis, MI, USA) Isopropanol (IPA, 99.5%, Sigma-Aldrich, Saint Louis, MI, USA), photoresist (AZ6112, Ruicai, Suzhou, China), and developing solution (2.38%, NMD-3, Ruicai, Suzhou, China), were used as received without further purification.

### 2.2. Metal Mesh Preparation

Firstly, glass substrates (2 × 2.5 cm^2^) were ultrasonically cleaned in deionized (DI) water, acetone, ethyl alcohol, and DI water for 20 min, respectively. Secondly, the nitrogen gun was used to dry the substrates, and spin-coat the positive photoresist on substrates by two-steps of 500 rpm for 5s and 4000 rpm for 30 s with the acceleration of 4000 rpm/s, followed by baking on a hot plate at 100 °C for 90 s to cure the photoresist. Thirdly, the substrates were naturally cooled and exposed for 2.3 s under a shadow mask and then developed for 60 s in the developing solution. Then, the samples were rinsed in flowing DI water and dried by nitrogen gun. Fourthly, an optical microscope was employed to check the defined patterns and the samples were transferred into the E-beam evaporation system and nickel/gold (Ni/Au) mesh of x nm/10 nm were deposited under a pressure below 5 × 10^−4^ Pa. After the metal mesh deposition, the samples were immersed in the acetone solution and lifted off by a low power ultrasonic bath for several ten seconds. The completed metal-mesh show a line width of 5 μm, and the active areas of 0.09 mm^2^ and 1 cm^2^.

### 2.3. PSCs Preparation and Characterization

At first, the prepared metal-mesh substrates were UV-ozone treated by 15 min, and the high conductive polymer PH 1000 were spin-coated on the metal mesh at 1000 rpm for 60 s and annealed on a hotplate at 150 °C for 15 min. Secondly, the PEDOT:PSS was spin-coated at 6000 rpm for 45 s and 150 °C annealing for 15 min. Thirdly, the MA_y_FA_1−y_PbI_x_Cl_3−x_ precursor solution was prepared by mixing PbI_2_, PbCl_2_ that was dissolved in DMF solution, and stirred for 2 h at 75 °C, the solution was then spin coated onto the PEDOT/PSS layer at 3000 rpm for 45 s; Mixing FAI, MAI in the solvent of DMF, stirring at room temperature until completely dissolved, then spin coated it onto the PbI_2_/PbCl_2_ layer at 3000 rpm for 45 s; after thermally annealing at 100 °C for 10 min the perovskite layer was formed. Fourthly, the PCBM (20 mg/mL in CB) was spin-coated on the perovskite layer at 2000 rpm for 40s, and the BCP (0.5 mg/mL in IPA) was spin-coated on the PCBM at 6000 rpm for 45s. Finally, the Ag (100 nm) electrode was thermally evaporated under a shadow mask and the fabricated devices (seeing Figures S1 and S2 in Supporting Information) have an active area of 0.09 cm^2^ and 1 cm^2^. As a reference, the ITO based PSCs, ITO/PEDOT:PSS/Perovskite/PCBM/BCP/Ag, were also fabricated under the same process conditions. 

The current density-voltage (J-V) characteristics were measured with a source measurement unit of Keithley 2400 and simulated AM1.5 G sun light (100 mW/cm^2^, SEN-EI Electric. Co. Ltd, XES-300T1, Osaka, Japan). The incident photo-to-current conversion efficiency (IPCE) was measured by the quantum efficiency measurement system (SCS10-X150, Zolix instrument. Co. Ltd, Beijing, ChinaZolix Instrument. Co. Ltd). The four-point-probe system was utilized to measure the sheet resistance of electrodes. The UV–visible spectrophotometer (Perkin-Elmer Lambda 950, Waltham, MA, USA) was used to characterize the transmittance spectra of different samples. The film morphology was characterized by a JSM-7800F extreme-resolution analytical field emission scanning electron microscope (SEM) (JEOL Ltd., Tokyo, Japan) and atomic force microscopy (AFM) (Agilent 5500, Santa Clara, CA, USA). Electrochemical impedance spectroscopy (EIS) measurements were performed on an electrochemical workstation (CHI600E, Shanghai Chenhua, Shanghai, China) with a 10 mV amplitude perturbation and frequencies between 100 Hz and 1 MHz. M-S plots were recorded on the same system under AC excitation amplitude of 30 mV at a frequency of 5 kHz. Transient photocurrent (TPC) measurement was performed with a system excited by a 532 nm (1000 Hz, 3.2 ns) pulse laser. Transient photovoltage (TPV) measurement was performed with the same system excited by a 405 nm (50 Hz, 20 ms) pulse laser. A digital oscilloscope (Tektronix, D4105, Beaverton, OR, USA) was used to record the photocurrent or photovoltage decay process with a sampling resistor of 50 Ω or 1 MΩ, respectively. All the measurements were performed under ambient atmosphere at room temperature.

## 3. Results and Discussion

**Figure 1:**
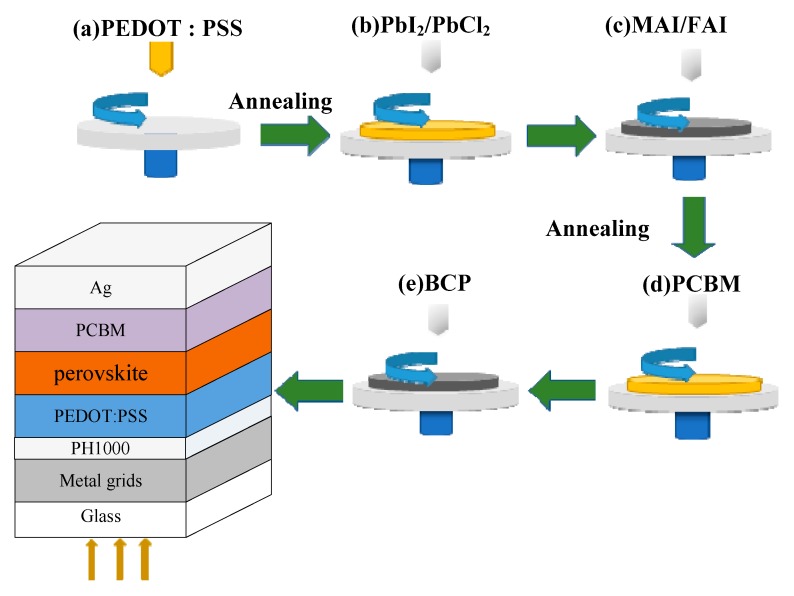
Process of indium thin oxide (ITO)-free perovskite solar cells (PSCs) based on a metal mesh transparent electrode.

The geometry of ITO-free planar PSCs with an inverted structure based on a metal mesh transparent electrode is shown in Figure 1. Here, the two-step solution processed MA_y_FA_1−y_PbI_x_Cl_3−x_ perovskite layer is chosen as the light absorber; the PEDOT:PSS and PCBM act as the hole and electron transport layers (HTL and ETL), respectively; the BCP is further used to modify the electron collection at the PCBM/Ag interface. In particular, to obtain optimal metal mesh with high transmittance and low resistance, the photolithography process was chosen to precisely define the line width and space between metal lines. Two curial interfaces related to the metal mesh were designed to improve the properties of Ni/Au transparent electrode, then the corresponding performance of ITO-free PSCs with small and large active areas are discussed as follows.

### 3.1. Optimization of Ni/Au Mesh Transparent Electrode

There are two curial interfaces introduced by the metal mesh, including the metal/substrate and metal/HTL interfaces. First, the good adhesion of metal mesh on substrate is necessary to fabricate the PSCs. Although the silver material possesses the lowest resistivity, it has been found in our previous work [36] that the adhesion of Ag mesh on glass substrates is relatively weak, and the mesh is easily damaged in the lift-off process, thus the Ni mesh is chosen to fabricate PSCs. However, the relatively low conductivity of Ni limits the improvement of device performance. In this work, an ultrathin Au is introduced to enhance the conductivity of Ni, and the shape and thickness of Ni/Au mesh has been further optimized. On the other hand, as the PEDOT:PSS HTLs (about 10 nm) is prepared by solution coating, the film quality is highly related to the smoothness of the underlying layer. Considering the thickness of the Ni/Au metal mesh has exceeded 50 nm, the high conductivity PHI000 (about 100 nm) is deposited to smooth the raised surface of the metal mesh, and simultaneously enhance the hole collection ability of Ni/Au electrode. It is noted that the PH1000 only causes a little loss of transmittance in the visible region [5], therefore the Ni/Au/PH1000 electrode is selected in the ITO-free PSCs.

**Figure 2:**
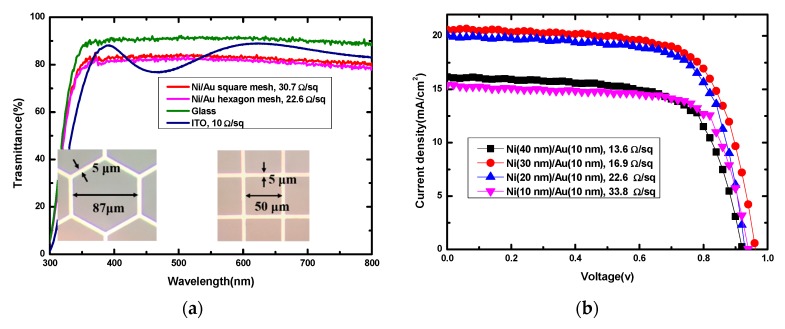
(**a**) Transmittance spectra of Ni (20 nm)/Au (10 nm) mesh (square, hexagon) and ITO on glass, and the glass substrate; (**b**) density-voltage (J-V) curves for PSCs with hexagon Ni(x nm)/Au (10 nm) (x = 10, 20, 30, 40 nm) metal mesh electrodes. The sheet resistances of Ni/Au and ITO electrode are also displayed in Figure 2.

Figure 2a provides the transmittance of spectra of Ni/Au mesh (square, hexagon) and ITO on glass, and the glass substrate. It can be seen that the glass shows the highest transmittance at over 90% in 300 nm to 850 nm, the commercial ITO coated glass substrate possesses an average transmittance over 80%, with the oscillation behavior related to the optical interference at the ITO/glass interface. For the Ni/Au meshes, although the highest transmittance is lower than the ITO sample, a relatively higher transmittance is obtained in the 300–370 nm and 420–500 nm regions. Their average transmittance has also exceeded 80% and the hexagon mesh shows a slightly low transmittance compared to the square mesh. Consequently, the optical performance of the Ni/Au mesh can meet the requirement of the transparent electrode. For the electrical property, the four-point-probe method is used to measure their sheet resistances. The corresponding values are also in Figure 2, showing that the hexagon mesh (22.6 Ω/sq) has lower resistance than the square mesh (30.7 Ω/sq), but larger resistance than that of the ITO (10 Ω/sq). From Figure S3, it is clear that the PSC with the Ni/Au square mesh electrode shows better performance compared to the device with a pure Ni electrode (58 Ω/sq); and the PSC with a hexagon Ni/Au electrode performs better than the device with a square Ni/Au electrode. Their improved PCEs mainly come from the increased J_SC_ values, which is in line with the results of sheet resistance. Thus, the hexagon Ni/Au mesh should be more suitable for fabricating ITO-free PSCs, and its thickness is further optimized. Here, the thickness of Au is fixed on 10 nm, and the thicknesses of Ni vary by 10 nm, 20 nm, 30 nm, and 40 nm. Table 1 shows the corresponding sheet resistances of the Ni/Au meshes. When the thickness of Ni increases from 10 to 40 nm, the sheet resistance values are 33.8 Ω/sq, 22.6 Ω/sq,16.9 Ω/sq and 13.6 Ω/sq, respectively. It is clear that the thicker the metal is, the lower the sheet resistance is. However, too thick a metal will lead to a worse lift-off effect, such as uneven edges of the metal wire and partial fracturing of the grid, which will induce a weak conductivity in the electrode. Meanwhile, if the thickness is less than 10 nm, the adhesion between Ni and glass substrate becomes very poor. Also, with metal deposited by electron beam evaporation it is difficult to form a homogeneous film at a thickness lower than 10 nm, as that will lead to a weaker conductivity [37]. Therefore, the thickness of Ni is a trade-off, which should be further determined by the device performance. As shown in Figure 2b and Table 1, all the devices show similar V_OC_ and FF values, and the J_SC_ dominants the overall PCEs. When the thickness of Ni is 10 nm or 40 nm, a lower PCE of about 10% is limited by the relatively poor J_SC_ values of 15.43 mA/cm^2^ and 16.16 mA/cm^2^. While if the 20 nm or 30 nm Ni is used, a J_SC_ exceeding 20 mA/cm^2^ can be achieved for PSCs, and the PSCs with30 nm Ni obtain superior PCE of 13.72%. As a result, the optimal metal mesh transparent electrode should be the hexagon Ni (30 nm)/Au (10 nm) mesh electrode, and the corresponding device performance is further investigated.

### 3.2. Performance of PSCs with Optimal Ni/Au Metal Mesh

**Figure 3:**
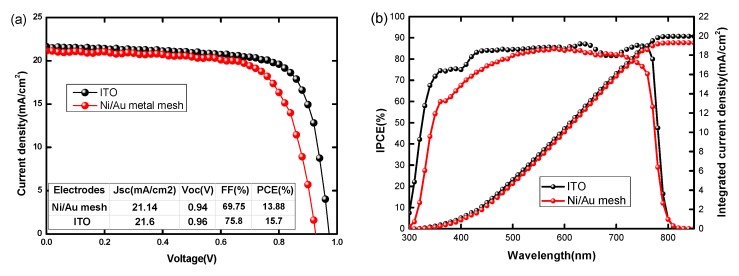

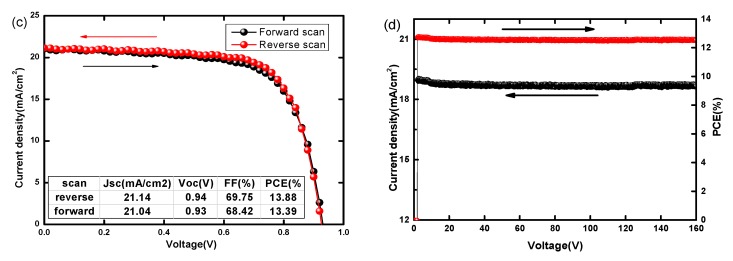
(**a**) J-V curves and (**b**) incident photo-to-current conversion efficiency (IPCE) spectra of PSCs based on Ni/Au mesh and PSCs based ITO measured under 100 mW/cm^2^ AM 1.5 G illumination; (**c**) J-V curves in forward and reverse scans and (**d**) steady output current density and PCE of PSCs based on Ni/Au mesh and the maximum power point (Vmax = 0.67 V).

Figure 3a presents the J-V curves of the champion PSC with the electrode and reference PSC with an ITO electrode. It can be seen that the PSC based on Ni/Au mesh obtains a champion PCE of 13.88%, V_OC_ of 0.94 V, J_SC_ of 21.14 mA/cm^2^, and FF of 69.75% at reverse voltage scan direction; while the forward scanned PCE is 13.39% with V_OC_ = 0.93 V, J_SC_ = 21.04 mA/cm^2^, and FF = 68.42%(Figure 3c). This negligible current hysteresis in the PSC based on Ni/Au mesh electrode is strongly related to the effects of PCBM passivation and BCP interface medication, which has been proved in our previous works [3,4]. Further, the steady-state outputs of current density and PCE at the maximum power point voltage of PSC based on Ni/Au mesh are shown in Figure 3d, the nearly unchanged current density and PCE outputs during 160 s illustrate the good operation stability of PSC. At the same time, the reference ITO based PSC shows a PCE of 15.70%, V_OC_ of 0.96 V, J_SC_ of 21.60 mA/cm^2^, and FF of 75.80%. Meanwhile, as shown in Figure S4, the lower leakage current and better rectification characteristics are in line with the relatively higher performance of PSC based on the ITO electrode. It is clear that the PSC based on Ni/Au mesh can obtain comparable PCE to that of the ITO based PSC, which demonstrates that the hexagon Ni/Au mesh is a promising ITO alternative. On the other hand, compared to ITO based PSC, the low V_OC_ and FF of Ni/Au based PSC may be explained by the relatively weak charge transport and collections ability. As J-V curves under AM 1.5 G illumination reveal the photovoltaic performance of PSC in the entire absorption range, to further study the photovoltaic conversion at single incident light wavelengths, the IPCE spectra are measured and the results are shown in Figure 3b. The integrated J_SC_ values of 19.29 mA/cm^2^ (Ni/Au-PSC) and 19.95 mA/cm^2^ (ITO-PSC) agree well with the measured J_SC_ in J-V measurements, which manifest the dependability of the J-V curve measurement. However, it should be noted that the IPCEs of ITO based PSC are higher than that of Ni/Au based PSC at each single wavelength from 300 nm to 800 nm. As the overall optical transmittance of Ni/Au and ITO are close, the perovskite film quality and the charge transport properties may account for the relatively low performance of the Ni/Au based PSC.

**Figure 4:**
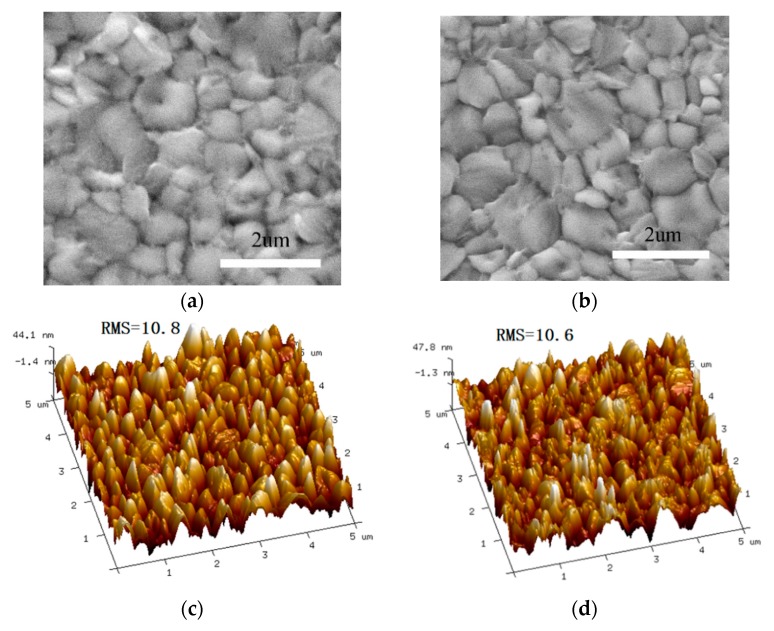
SEM and AFM images of perovskite film based on (**a**,**c**) Ni/Au mesh and (**b**,**d**) ITO electrodes. The unit of RMS values are nm.

**Figure 5:**
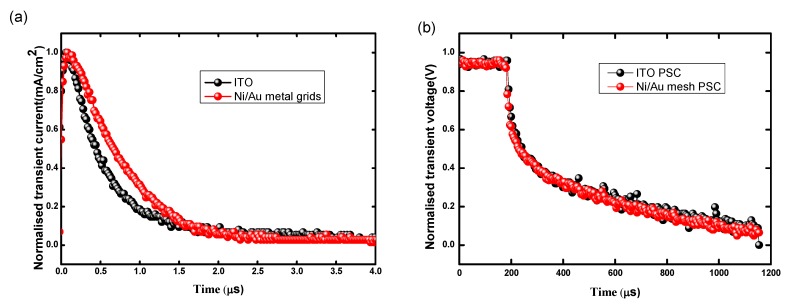
(**a**) Transient photocurrent (TPC) and (**b**) transient photovoltage (TPV) decay curves of the PSCs based on Ni/Au mesh and ITO electrodes.

First, as the same preparation method of perovskite film has been evaluated in our previous work [3], in the present work the primary concern is the morphology of perovskite films. Figure 4a,c and Figure 4b,d show the SEM and AFM images of perovskite film based on Ni/Au mesh and ITO electrodes. It has been observed that both the perovskite films are compact and smooth, there is no significant difference in the grain sizes and root-mean-square roughness (RMS) values. From this it can be understood that the PH1000 buffer layer has smoothened the raised surface of Ni/Au mesh, which ensures a similar film morphology of perovskite on PEDOT:PSS/PH1000/Ni/Au/Glass and PEDOT:PSS/ITO/Glass samples. Here, we extracted the series resistance (R_s_) and shunt resistance (R_sh_) from the J-V curves by the parameter extraction method in our previous work [38]. As show in Figure S5, the experimental data are well reproduced by the fitting curves and the corresponding R_s_, R_sh_, ideality factor, saturation current are listed in Table S1. It is clear that the PSC based on Ni/Au mesh show a R_S_ of 1.2 Ω·cm^2^ and R_SH_ of 5.548 kΩ·cm^2^, while that of the ITO based PSC are 1.0 Ω·cm^2^ and 6.398 kΩ·cm^2^, combining the larger ideality factor and saturation current values, thus the more efficient carrier transport contributes to the better performance of ITO based PSC. To further study the charge transport properties, transient photocurrent (TPC) and transient photovoltage (TPV) measurements are carried out. The TPC can reflect extraction and transport property of carriers, and the TPV provides insight to carrier recombination property in a solar cell. As shown in Figure 5a, the ITO based PSC has a relatively faster photocurrent decay (0.46 μs) compared with the Ni/Au based PSC (1.96 μs), suggesting that the extraction and transport of carriers are more efficient in ITO based PSC. Meanwhile, the photovoltage decay processes are displayed in Figure 5b, the fitted charge recombination lifetimes are 1389.04 μs and 1183.34 μs for PSC based on ITO and the Ni/Au mesh, respectively. What is more, EIS measurements were carried out to evaluate the carriers’ recombination in the corresponding PSCs. The corresponding Nyquist plots, the equivalent circuit diagram, and fitted results are shown in Figure S6 and Table S2. It can be found that, the PSC based on Ni/Au mesh achieved a relatively smaller R_rec_ (carrier recombination resistance) and shorter electron lifetime. Simultaneously, the sheet resistance of Ni (30 nm)/Au (10 nm) mesh (16.9 Ω/sq) is higher than that of ITO. Combining the results of AFM, SEM, TPC, TPV, and conductivity, the relatively low performance of PSC based on Ni/Au mesh can be understood.

**Figure 6:**
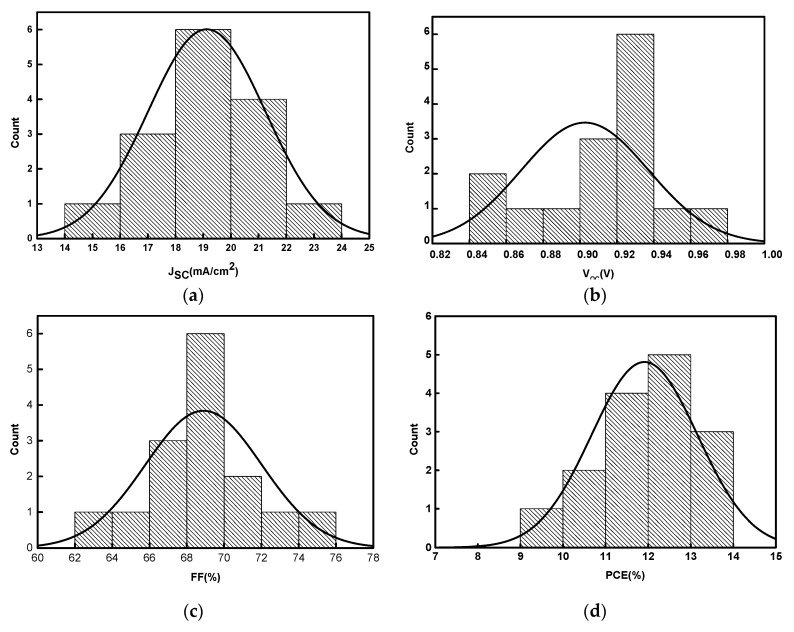
Histograms of photovoltaic parameters. (**a**) J_SC_, (**b**) V_OC_, (**c**) FF, and (**d**) PCE for PSC based on Ni/Au mesh electrode. The histograms shown are the photovoltaic parameters for 15 devices.

**Figure 7:**
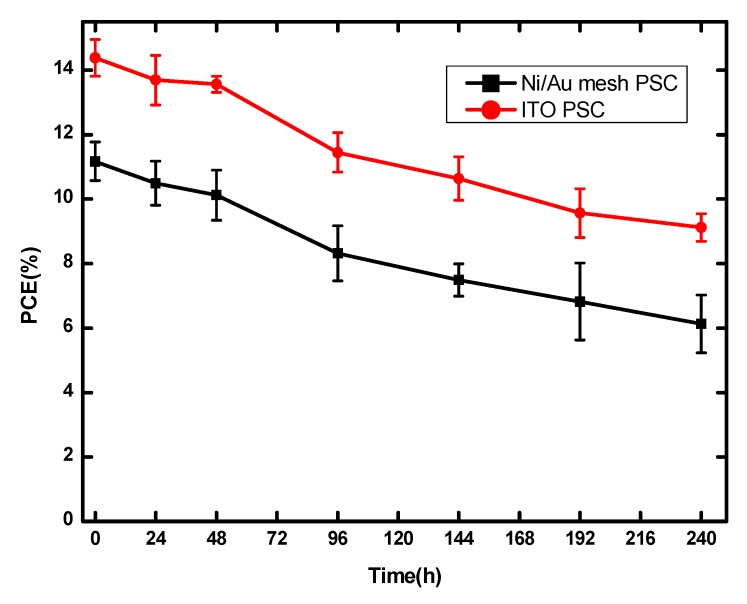
Storing stability of Ni/Au mesh and ITO based PSCs without encapsulation in N_2_ for 240 h.

In addition, the statistical results of photovoltaic parameters and the store stability of Ni/Au based PSCs are shown in Figure 6 and Figure 7, the device number in the statistical analysis is 15. From Figure 6, it can be seen that most of the V_OC_ values concentrate in the range from 0.84 V to 0.98 V and eight of them exceed 0.92 V; the J_SC_ ranges from 14 mA/cm^2^ to 24 mA/cm^2^, with eight of them higher than 19 mA/cm^2^; the FF from 62% to 76%, with nine of them larger than 69%; the PCE from 9% to 14% with eight of them exceeding 12%. All the photovoltaic parameters exhibit Gaussian distribution, which suggests the good reproducibility of Ni/Au based PSCs. Here, their FF and V_OC_ values should be further improved to obtain high performance PSCs. Besides the optimization of Ni/Au mesh electrode, more efficient charge transport layers [3,39], and interface passivation [1,40] are feasible approaches to achieve this goal. Furthermore, from Figure 7, the PSCs based on Ni/Au mesh and ITO present a similar trend of PCE degradation. After being stored in N_2_ for 240 h, the Ni/Au based PSCs keep 58% of their initial PCE values, and the ITO based devices maintain 60% of their initial PCE values. Therefore, the PSCs based on Ni/Au mesh possess comparable photovoltaic performance and stability to the reference ITO based PSCs, and the Ni/Au mesh is one of the promising ITO alternatives to fabricate PSCs. Nowadays, owing to the improvement of perovskite quality and interface modification, the PCE of planar ITO based PSCs has exceeded 23% [1]. It is believed that there is plenty of room for performance improvement of PSCs based on metal mesh transparent electrodes, which will be further investigated in our future works.

**Figure 8:**
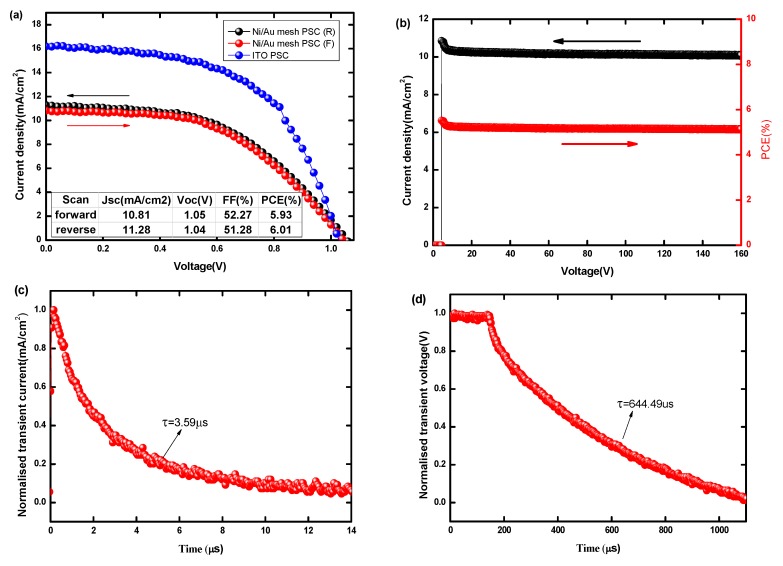
(**a**) J-V curves in forward and reverse scans for large-area PSCs based metal mesh, (**b**) steady output characteristics curve, (**c**) TPC and (**d**) TPV decay curves.

What is more, the large-area (1 cm^2^) Ni/Au mesh-based PSCs are fabricated under the same process conditions as the small-area (0.09 cm^2^) PSCs. Figure 8a,b shows the J-V curve, photovoltaic parameters, and steady outputs of large-area Ni/Au based PSCs and ITO based reference PSCs. The Ni/Au based PSC obtains a PCE of 6.01%, with V_OC_ = 1.04 V, J_SC_ = 11.28 mA/cm^2^, and FF = 51.28% at the reverse voltage scan direction, as well the steady PCE and current density outputs under continuous AM 1.5 G illumination during 160 s. The forward scanned PCE = 5.93%, V_OC_ = 1.05 V, J_SC_ = 10.81 mA/cm^2^, and FF = 52.27%, thus the current hysteresis effect is negligible. While the ITO based PSC shows a higher PCE of 9.01% with V_OC_ = 1.02 V, J_SC_ = 16.24 mA/cm^2^, and FF = 55.59%, it should be noted that the J_SC_ and FF of large area PSCs are obviously lower than the small area PSCs (Figure 3), which is due to relatively poor charge extraction and strong charge recombination related to the more defects and traps in the larger-area perovskite layer. This can be partly proved by the TPC and TPV results. As shown in Figure 8c,d, the large-area PSC displays a slower photocurrent decay (3.59 μs) compared to the small-area cell (1.96 μs), suggesting inefficient charge transport and extraction in the large-area cell. Meanwhile, a faster photovoltage decay (644.49 μs) than the small-area one (1183.34 μs) reveals the relatively strong charge recombination in the large-area cell. Therefore, the perovskite film quality and interface modification are crucial to further improving the performance of large-area devices. Considering the drawbacks of ITO, the meal mesh based large-area PSCs may find wider applications in the near future.

## 4. Conclusions

In summary, we carefully designed and deposited hexagon Ni/Au metal mesh transparent electrodes with high transmittance and low resistance by photolithography and the e-beam evaporation process. To be an efficient electrode for PSCs, the Ni was used to improve the adhesion of metal mesh to glass substrate and the Au was used to increase the conductivity of the metal mesh. The conductive polymer PH1000 not only smooths the raised surface of metal mesh but also enhances the charge collection ability of metal mesh. The optimal hexagonal Ni (30 nm)/Au (10 nm)/PH1000 electrodes were employed to fabricate ITO-free PSCs. The champion PSC (0.09 cm^2^), with a typical planar structure, obtained a PCE of 13.88%, negligible current hysteresis, steady current density and PCE outputs, good repeatability and storing stability. The comparable performance to the ITO based reference PSC demonstrates that the Ni/Au mesh transparent electrodes are a promising ITO alternative to fabricate efficient PSCs. Further, we tried to fabricate the large area (1 cm^2^) devices under the same low-temperature process, and achieved a PCE over 6% with negligible hysteresis and stable steady outputs. And the perovskite film quality and interface modification are crucial to further improving the performance of large-area devices. Considering the drawbacks of ITO, the meal mesh-based PSCs may find wider applications in the near future.

## Figures and Tables

**Table 1:** Photovoltaic parameters of PSCs with various Ni(x nm)/Au (10 nm) meshes.

Ni Thickness (nm)	Ni/Au Resistance (Ω/sq)	J_SC_ (mA/cm^2^)	V_OC_ (V)	FF (%)	PCE (%)
40	13.6	16.16	0.90	68.76	10.13
30	16.9	20.67	0.96	69.09	13.72
20	22.6	20.04	0.92	71.22	13.14
10	33.8	15.43	0.94	71.68	10.43

## Supplementary Materials

The following are available online at https://www.mdpi.com/2079-4991/9/7/932/s1, Figure S1: Photos of ITO-free PSCs (0.09 cm^2^) based on Ni/Au mesh electrode (a) light incident surface (b) backlight surface, Figure S2: Photos of large-area ITO-free PSCs (1 cm^2^) based on Ni/Au mesh electrode (a) light incident surface (b) backlight surface., Figure S3: J-V curves of ITO-free PSCs (0.09cm^2^) based on Ni (30 nm) square, Ni(20 nm)/Au(10 nm) square, and Ni(20 nm)/Au(10 nm) hexagon mesh electrodes. The sheet resistance of pure Ni and Ni/Au meshes are about 58 Ω/sq, 30.7 Ω/sq, and 22.6 Ω/sq, thus the PSC with Ni/Au hexagon electrode obtains higher Jsc, FF, and PCE values, Figure S4: Semi-log plots of dark JV curves for PSC based on Ni/Au mesh and ITO electrodes, Figure S5: JV curves under AM 1.5G illumination for PSC based on Ni/Au mesh and ITO electrodes. Here the symbols represent the experimental data and the solid lines indicate the fitting curves, Figure S6: Nyquist curves of PSCs based on Ni/Au mesh and ITO electrodes. The insert is the equivalent circuit for the fittings, where R_s_ represents the series resistive elements related to connections and devices, R_rec_ the carrier recombination resistance and, C_PE_ the constant phase element.; Table S1: R_s_, R_sh_, saturation current, and ideality factor extracted from JV curves under AM 1.5G illumination, Table S2: EIS parameters extracted from Nyquist curves. The electron lifetime is the reciprocal of the frequency of the maximum point of the semi-circular response.
