# Supplementary material for: Efficient Ni/Au Mesh Transparent Electrodes for ITO-Free Planar Perovskite Solar Cells
Source: Nanomaterials (Basel). 2019 Jun 28;9(7):932. doi: 10.3390/nano9070932 (PMC6669768; doi:10.3390/nano9070932)
Supplement: Supplementary file 1 [file nanomaterials-09-00932-s001.pdf]

Supporting Information

Efficient Ni/Au mesh transparent electrodes for ITO-free planar perovskite solar cells

Dazheng Chen*[,a,b†], Gang Fan[a], Hongxiang Zhang[b] , Long Zhou[b], Weidong Zhu[a], He Xi[a,c], Hang Dong[a], Shangzheng Pang[a], Xiaoning He[a], Zhenhua Lin[a], Chunfu Zhang*[,a,b], Jincheng Zhang[a,b], Yue Hao[a]

[a]Wide Bandgap Semiconductor Technology Disciplines State Key Laboratory, School of Microelectronics, Xidian University, Xi'an, 710071, China

[b]Shaanxi Joint Key Laboratory of Graphene, Xidian University, Xi'an, 710071.

[c]School of Advanced Materials and Nanotechnology, Xidian University, Xi'an, 710071, China

Corresponding Auhtor: dzchen@xidian.edu.cn, cfzhang@xidian.edu.cn

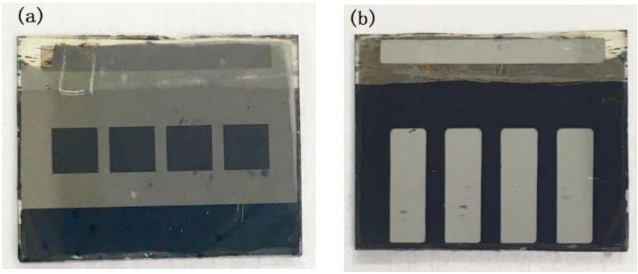

Fig. S1 Photos of ITO-free PSCs (0.09 cm^2) based on Ni/Au mesh electrode (a) light incident surface (b) backlight surface.

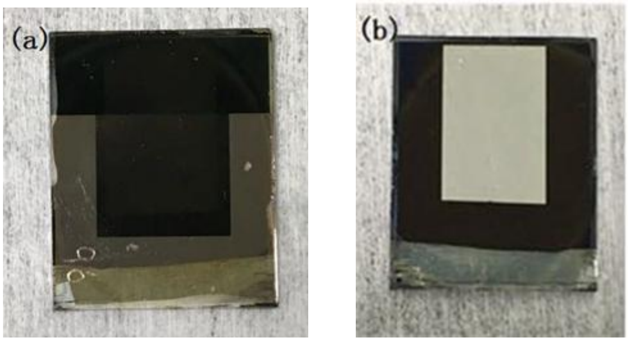

Fig. S2 Photos of large-area ITO-free PSCs (1 cm^2) based on Ni/Au mesh electrode (a) light incident surface (b) backlight surface.

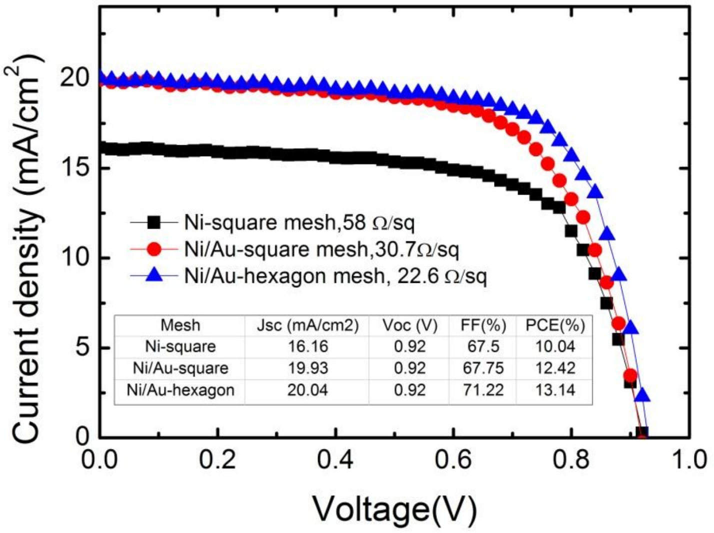

Fig. S3 J-V curves of ITO-free PSCs (0.09cm^2) based on Ni (30 nm) square, Ni(20 nm)/Au(10 nm) square, and Ni(20 nm)/Au(10 nm) hexagon mesh electrodes. The sheet resistance of pure Ni and Ni/Au meshes are about 58 Ω/sq, 30.7 Ω/sq, and 22.6 Ω/sq, thus the PSC with Ni/Au hexagon electrode obtains higher Jsc, FF, and PCE values.

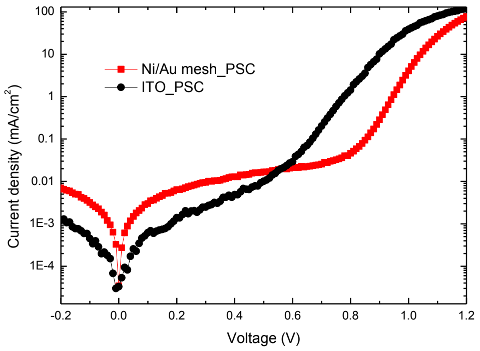

Fig. S4 Semi-log plots of dark JV curves for PSC based on Ni/Au mesh and ITO electrodes.

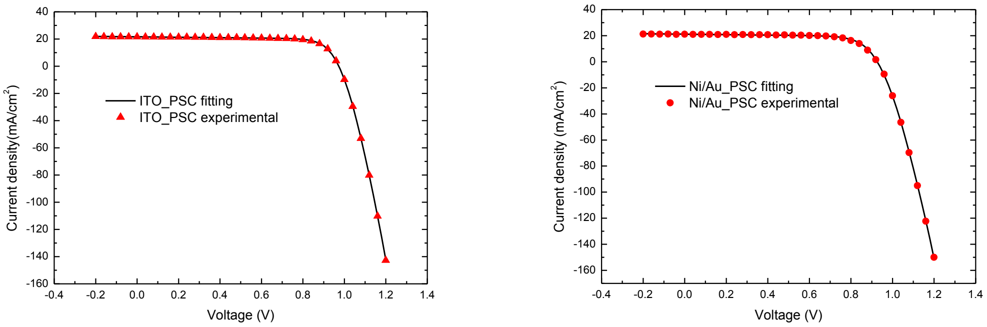

Fig. S5 JV curves under AM 1.5G illumination for PSC based on Ni/Au mesh and ITO electrodes. Here the symbols represent the experimental data and the solid lines indicate the fitting curves.

Table S1. R_s, R_{sh}, saturation current, and ideality factor extracted from JV curves under AM 1.5G illumination.

Device	R_s (Ωcm^2)	R_{rec} ($k\Omega cm^2$)	Saturation current (mA/cm^2)	Ideality factor
ITO_PSC	1.0	6.398	4.2729×10^{-9}	2.0756
Ni/Au mesh_PSC	1.2	5.548	6.1593×10^{-7}	1.6903

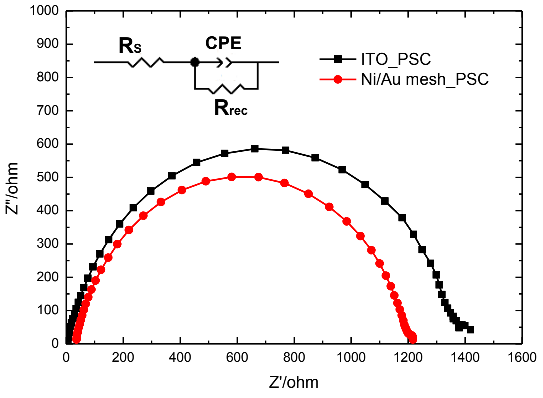

Fig. S6. Nyquist curves of PSCs based on Ni/Au mesh and ITO electrodes. The insert is the equivalent circuit for the fittings, where R_s represents the series resistive elements related to connections and devices, R_{rec} the carrier recombination resistance and, C_{PE} the constant phase element.

Table S2. EIS parameters extracted from Nyquist curves. The electron lifetime is the reciprocal of the frequency of the maximum point of the semi-circular response.

Device	R_s (Ωcm^2)	R_{rec} ($k\Omega cm^2$)	Electron lifetime(μs)
ITO_PSC	5.8	1.437	84.6
Ni/Au mesh_PSC	7.6	1.145	68.0
